# The developmental origins of moral concern: An examination of moral boundary decision making throughout childhood

**DOI:** 10.1371/journal.pone.0197819

**Published:** 2018-05-29

**Authors:** Karri Neldner, Charlie Crimston, Matti Wilks, Jonathan Redshaw, Mark Nielsen

**Affiliations:** 1 School of Psychology, University of Queensland, Brisbane, Queensland, Australia; 2 Department of Psychology, University of Johannesburg, Auckland Park, South Africa; University of L'Aquila, ITALY

## Abstract

Prominent theorists have made the argument that modern humans express moral concern for a greater number of entities than at any other time in our past. Moreover, adults show stable patterns in the degrees of concern they afford certain entities over others, yet it remains unknown when and how these patterns of moral decision-making manifest in development. Children aged 4 to 10 years (*N* = 151) placed 24 pictures of human, animal, and environmental entities on a stratified circle representing three levels of moral concern. Although younger and older children expressed similar overall levels of moral concern, older children demonstrated a more graded understanding of concern by including more entities within the outer reaches of their moral circles (i.e., they were less likely to view moral inclusion as a simple in vs. out binary decision). With age children extended greater concern to humans than other forms of life, and more concern to vulnerable groups, such as the sick and disabled. Notably, children’s level of concern for human entities predicted their prosocial behavior. The current research provides novel insights into the development of our moral reasoning and its structure within childhood.

## Introduction

Early humans lacked the knowledge, time and resources to consider the welfare of entities beyond their direct kin and ingroups. Over recent generations, however, societies have begun to extend moral rights to an increasingly broader array of human groups, as well as towards certain non-human animals and elements of the natural environment [1]. Singer [2] uses the metaphor of an expanding ‘moral circle’ to denote this growing boundary that distinguishes between entities worthy and unworthy of moral consideration. In adults at least, stable patterns persist in the relative degrees of concern afforded towards certain entities over others [3]. Moral consideration can be defined as the acknowledgement that an entity has inherent moral standing, tied to a perceived moral obligation and commitment to actively defend their well-being [3]. Family, friends and in-group members sit at the very center of our moral circles, whereas various outgroup members are kept relatively distal. Non-human animals and environmental entities typically sit at the periphery, whereas villains are cast out altogether, and afforded no moral recognition [3].

While the normative structure of adults’ moral circles has been established [3], it remains unknown when this structure manifests in development. Further, while our moral boundary decision making can be flexible and context dependent [4–7], there appears to be no change in the overall levels of moral expansiveness across age in adults [3]. Crucially, the limits of children’s moral boundaries throughout development have not been examined. The current research thus charts the origins of morally expansive decision making, and how patterns of moral concern might emerge and change throughout childhood. This research is the first to address children’s moral reasoning at an abstract level using various distal, hypothetical agents as opposed to proximal characters (used heavily in the narrative-based scenarios of the existing literature), providing greater scope on the relative patterns of concern afforded across a range of entities within a socially neutral context.

There is extensive existing research exploring moral development and reasoning throughout childhood. For example, there is evidence for basic prosocial preferences emerging from as young as three months [8,9]. Children act prosocially early in development [10,11] and increasingly engage in higher-cost prosocial behavior as they enter middle childhood (around eight years; see [12] for review). Crucially, high (but not low) cost prosocial behavior has been linked to moral reasoning ability [13,14], suggesting that moral reasoning becomes more considered/nuanced with age [15]. A large body of literature also demonstrates that, as children age, they become increasingly capable of considering social and group factors in conjunction with moral concern when making judgements about resource allocation [16] and social exclusion [16,17]. This higher level moral reasoning is also reflected in behavior. Children are shown to understand sharing norms from four years, but do not engage in equal sharing themselves until around eight years [18].

The use of vignettes [19,20] or hypothetical scenarios involving individual characters [18] is typical of the existing literature (e.g., asking children what they think about a group member who violated a particular group norm). As such, these moral scenarios are embedded within a narrative that might itself individualize the character to the child, thus influencing the associated moral judgments made. This limits our capacity to understand children’s perceptions of a range of entities outside of their direct and momentary experience. Moreover, the use of complex vignettes has largely limited the capacity of the development field to conduct research with young children, and in line with this, many studies of moral reasoning focus exclusively on older children [19,21].

In the current research, we employ a literal, visuospatial analogue of Singer’s [2] ‘moral circles’ metaphor [3,22] to investigate the development of children’s moral concern. Children aged 4 to 10 years were invited to place pictures of a range of target entities–both human and non-human–on a stratified circle representing three levels of moral concern. Although older children demonstrate more nuanced moral reasoning skills, and more prosocial behaviors in general than younger children [18,20,23,24], past research has failed to find any association between age and moral expansiveness in adults [3]. Therefore, we made no specific predictions regarding age-related changes in moral expansiveness across the developmental trajectory.

Past research has shown that moral expansiveness maps onto prosocial behavior in adults [3]. In line with this, we predicted that children who are more morally expansive (i.e., consider more entities worthy of moral concern) would donate more of a desirable resource than those less morally expansive. Finally, based on Crimston and colleagues’ work [3], we predicted that children would hold more moral concern for humans versus other forms of life, objects in comparison to villains, high versus low sentience animals [25], family members compared to ingroup members, and revered versus needy entities. Based on other previous literature, we further predicted that children would hold moral concern for ingroup versus outgroup members [26,27], and pets versus food animals [28].

## Materials and methods

### Participants

One hundred and fifty-one children (*M* = 7.02 years, *SD* = 1.95 years; 72 males, 79 females; range = 4–10 years) were recruited from a large science museum in a metropolitan city. Participants included 19 four-year-olds, 21 five-year-olds, 25 six-year-olds, 21 seven-year-olds, 22 eight-year-olds, 24 nine-year-olds, and 19 10-year-olds. Post-hoc power analyses indicated that this sample size provided a greater than 90% chance of detecting a medium size age effect. As GLMM is a regression model, the power analysis was based on Cohen’s [29] conventional figures for detecting a medium size correlation (i.e., our sample of 151 exceeded the required 85 participants to have an 80% chance of detecting a medium size relationship [r = .30] between age and moral expansiveness). An additional five children were excluded from analyses due to shyness (*N* = 2), or failure to pass comprehension checks (*N* = 3). While specific demographic information was not collected, previous unpublished data conducted at the science museum indicates that participating families are from middle-class socioeconomic backgrounds and the majority identify as Caucasian. Signed parental consent was obtained and the study approved by the School of Psychology Student Research Review Committee at the University of Queensland (clearance number: 15-PSYCH-PHD-64-AH).

### Procedure

**Fig 1:**
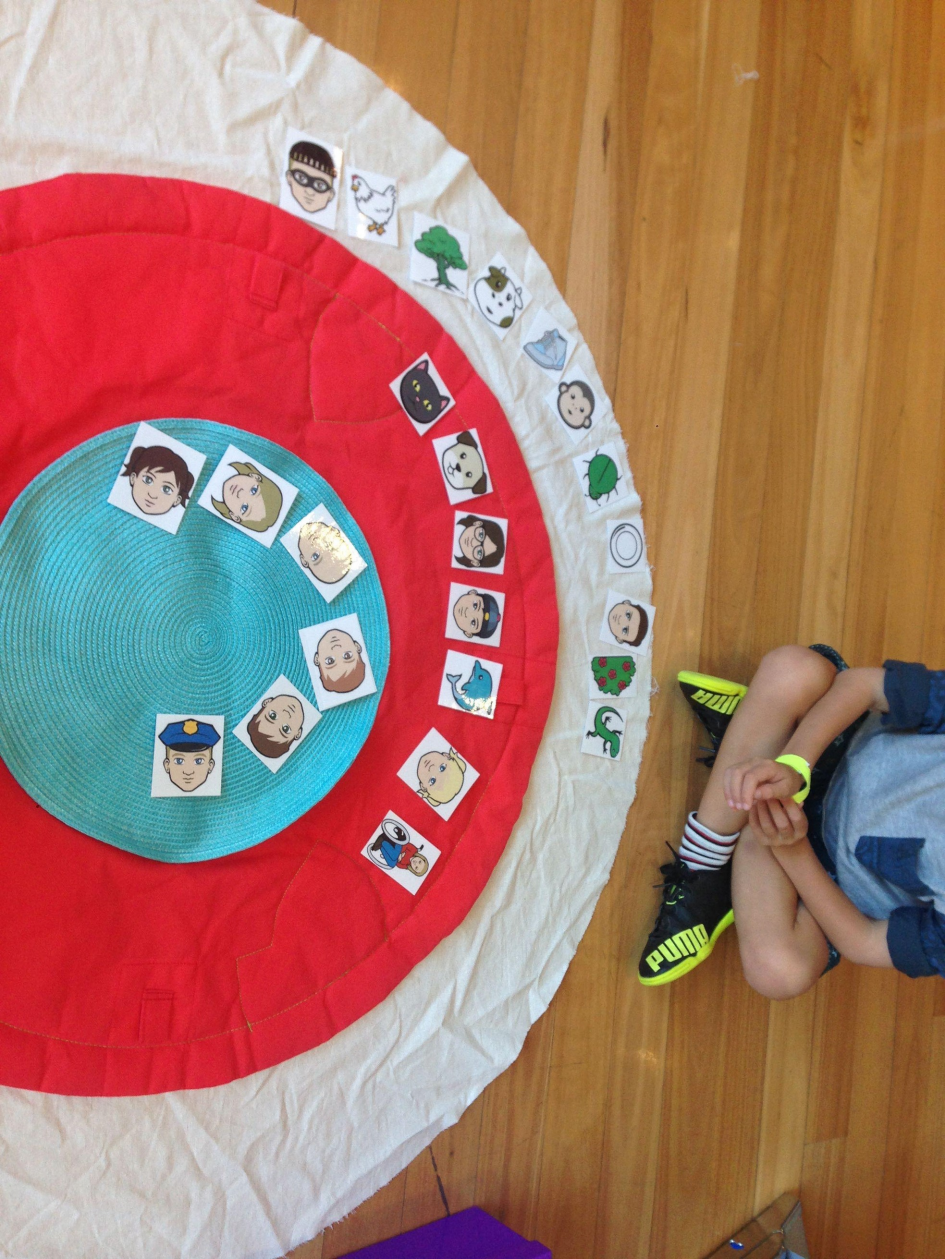
The experimental setup used throughout testing.

Children were recruited in the foyer of the science museum and invited to play a 5-10-minute game. Children were seated next to the testing mat, which consisted of three circles: an inner blue circle, a secondary red circle and an outer white circle (see Fig 1). The experimenter then revealed each of the 24 pictures depicting the entities for the study (these were shuffled before each participant to ensure random presentation). The entity items spanned 12 categories: family & friends, ingroup, outgroup, revered, needy, villains, high-sentient animals, low-sentient animals, food animals, pets, plants, and objects (categories were selected based on original Moral Expansiveness Scale (MES) entities used in Crimston and colleagues’ study (2016) [3], with additional entities relevant to the current sample included based on planned hypotheses; refer to S1 File for stimuli). The child was asked to name each picture as it was shown; if the child could not identify the picture, that entity was omitted from testing for that child (this occurred rarely at just over 5% of children). For the entities that had a specific descriptor, such as a child from Australia or China, the experimenter first labeled the picture, then asked if the child was aware of such an entity. The target was omitted if children were not familiar with its descriptor. The experimenter explained that the child was going to place all the pictures on the mat. Entities that the child really cared about could be put into the inner circle; those they cared about a little bit could be put into the middle circle; and entities that they did not care about at all could be put into the outer circle. The experimenter held up each picture and asked the child “how much do you care about [the entity]? Where do you want to put [the entity]?” The child was then invited to place the picture on the mat. A comprehension check determining children’s recall about what each circle represented was conducted before placing the first pictures, and again between each set of six pictures to ensure that children could accurately identify the meaning of each circle. Overall, recall was consistently accurate, with only 3 children removed due to an inability to correctly recall the circle categories.

After the game, the experimenter told the child that they would receive six stickers as a reward for doing a good job. She explained that the child could keep as many of the stickers as they wanted, but that there were some children coming in later who might not get any stickers, and that–if the child wanted–they could give some stickers away to those children. She provided a donation box and an envelope and reiterated that the child could give away as many of the 6 stickers as they liked by putting them in the box, and keep as many stickers as they liked by putting them in the envelope. The experimenter covered her eyes with her hands and asked the child to tell her when they had finished organizing their stickers.

## Results

### Moral concern

Children’s responses on the main task were entered into a series of generalized linear mixed model (GLMM) analyses using the GLIMMIX procedure in SAS 9.3 [30]. Given no directional hypotheses were proposed regarding qualitative shifts in children’s moral circles at specific ages, it was determined that age would be entered into the analyses as a continuous predictor. The 3-level dependent variable was classified as ordinal, and the models were therefore tested against a multinominal distribution with a cumulative logit link function. A number of models were tested, each containing a unique combination of fixed effects nested within the following full factorial model: Age (continuous variable ranging from 4–10) x Gender (boys vs. girls) x Entity (24 entities). Comprehensive details regarding the model selection process can be found in the Supporting Information (S1 File).

**Fig 2:**
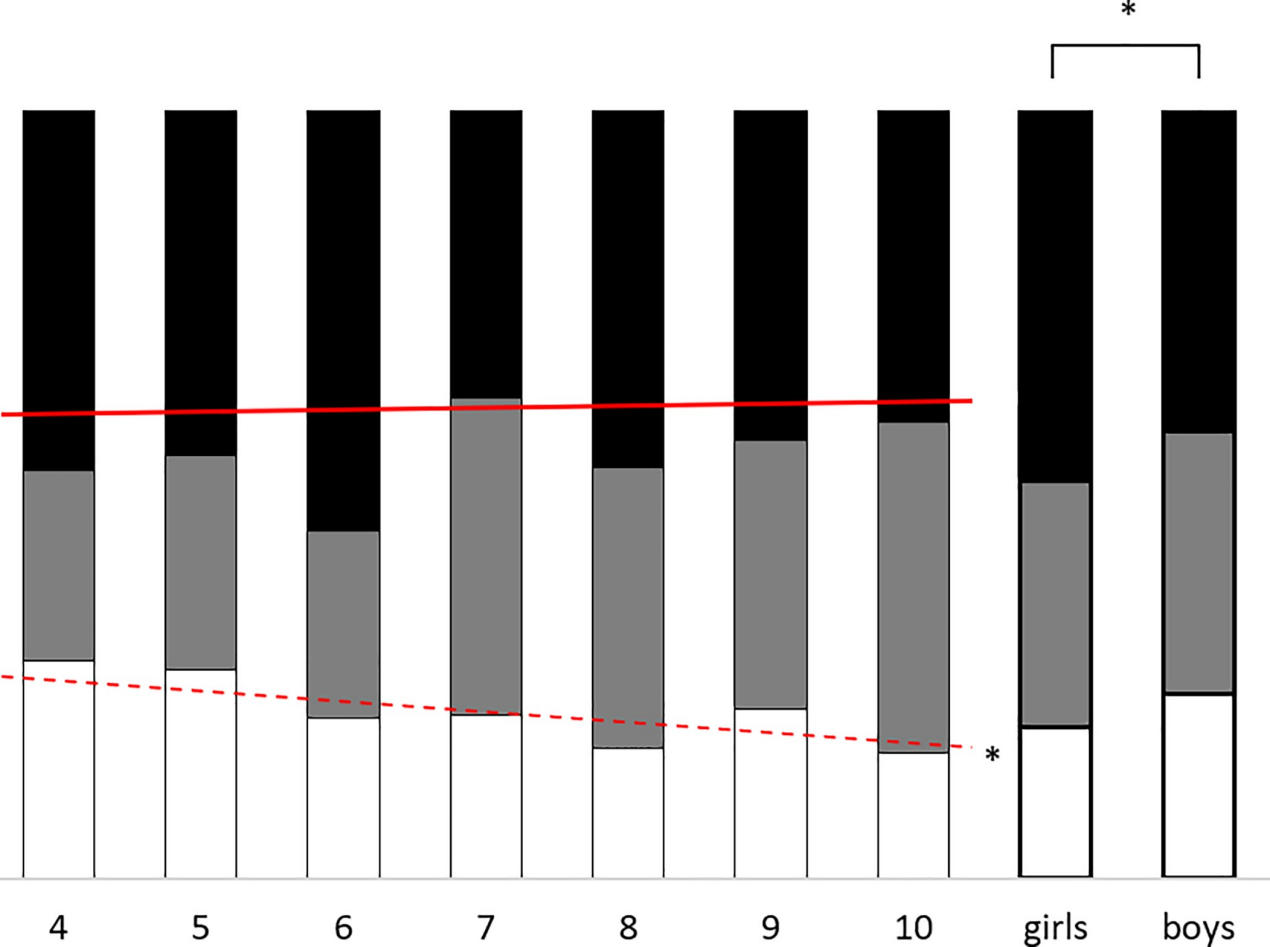
Summary of children’s overall levels of moral concern (collapsed over all 24 entities) across age groups and gender. White bars indicate the proportion of ‘not caring at all’ responses; grey bars indicate the proportion of ‘caring a little’ responses, and black bars indicate the proportion of ‘caring a lot’ responses. The solid red line indicates the (non-significant) linear effect of age. The dashed red line indicates the significant linear effect of age (*p* < .001) on children’s ‘not caring at all responses’ (compared to the other two responses combined). Across entities, girls cared significantly more than boys (*p* = .013).

The best fitting model (AIC = 6005.48) contained significant fixed effects of Gender and Entity, as well as a significant Age x Entity interaction and a significant random intercept. The best fitting model did not contain a main Age effect, and when forced into the model this effect did not reach significance, *F* (1, 3415) = 0.09, *p* = .768. Thus, there was no evidence that children’s overall level of moral concern changed as they became older (i.e., there was no overall age-based moral expansiveness; see Fig 2 for a summary of descriptive data). Instead, the Age x Entity interaction, *F* (23, 3415) = 3.36, *p* < .001, which qualified the Entity effect, *F* (23, 3415) = 5.09, *p* < .001, suggests that children cared about *different entities* as they got older. The Gender effect, *F* (1, 3415) = 6.15, *b* = .38, SE = .15, *p* = .013, suggests that girls showed a higher level of moral concern than boys across all entities included in the task. The random intercept, *b* = .70, SE = .11, *p* < .001, indicates individual variation in overall levels of moral concern.

### Post hoc binomial analysis

Although the *a priori* cumulative logistic analysis failed to uncover evidence for overall age-based moral expansiveness, the descriptive data told a more nuanced story. Specifically, younger children gave a much higher proportion of ‘not caring at all’ responses than older children (see Fig 2). We therefore conducted a further series of *post hoc* binomial GLMMs with a 2-level dependent variable: (i) ‘not caring at all’, and (ii) ‘caring at least a little’ (which encompassed responses of ‘caring a little’ and ‘caring a lot’). The best performing of these models (AIC = 2580.55) included Gender, Entity, and Age x Entity effects as before, but it also included an additional significant main effect of Age, *F* (1, 3415) = 14.30, *p* < .001. As children get older, they are more likely to care at least a little about more entities, *b* = .23, SE = .06. Thus, the *post hoc* binomial analysis provides strong evidence for one form of age-based moral expansiveness. Failure to detect this effect in the cumulative logistic GLMM was likely due to the fact that younger children’s responses were less likely to give the intermediate response. In other words, younger children were more likely to indicate liking entities a lot *and* not at all, with these effects cancelling each other out and resulting in similar average scores to older children.

### Age-based changes in the entities of moral concern

**Fig 3:**
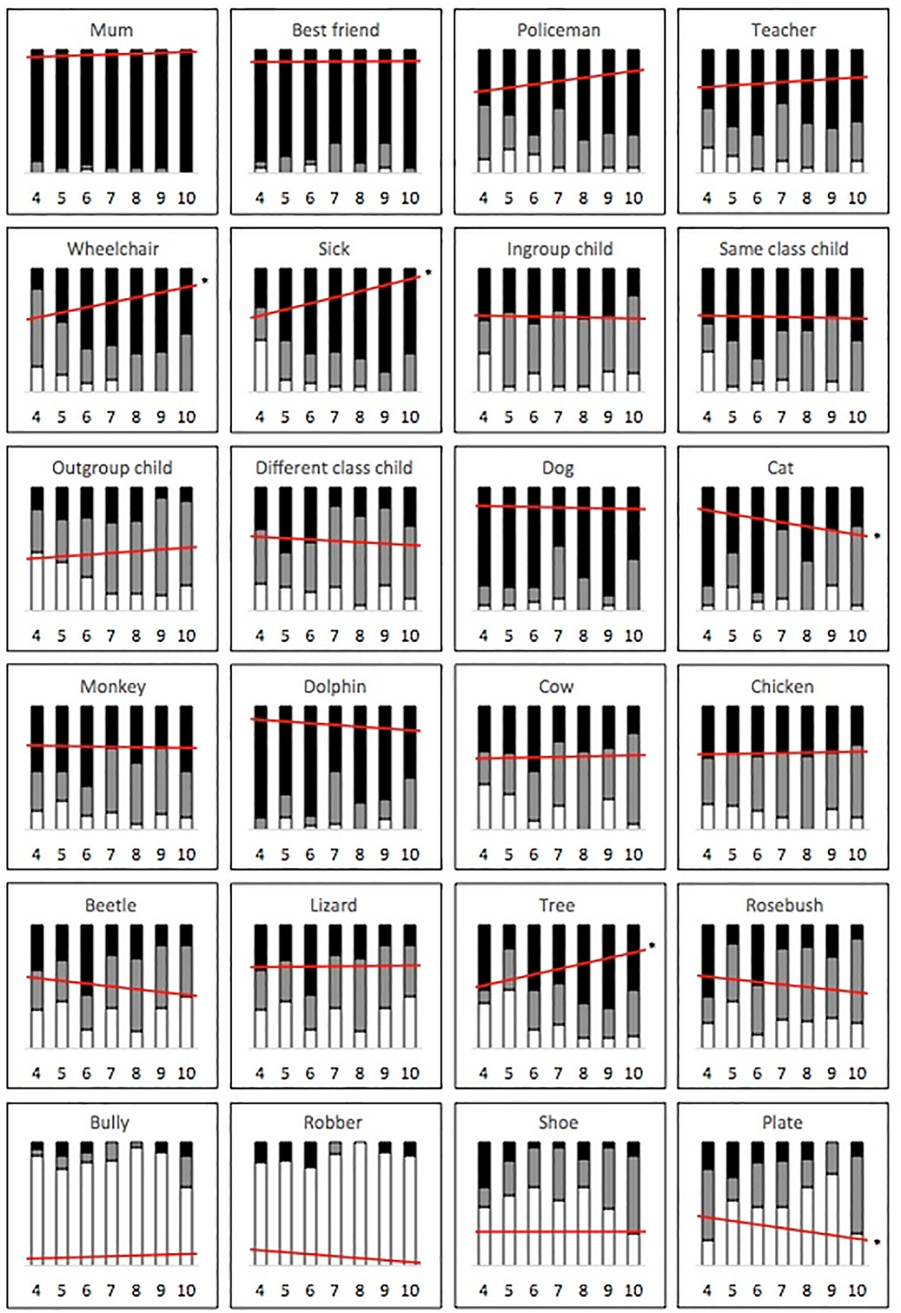
Summary of children’s levels of moral concern towards each entity across age groups. White bars indicate the proportion of ‘not caring at all’ responses; grey bars indicate the proportion of ‘caring a little’ responses, and black bars indicate the proportion of ‘caring a lot’ responses. Red lines indicate linear effects of age. Targets with significant age effects are marked with an asterisk.

**Fig 4:**
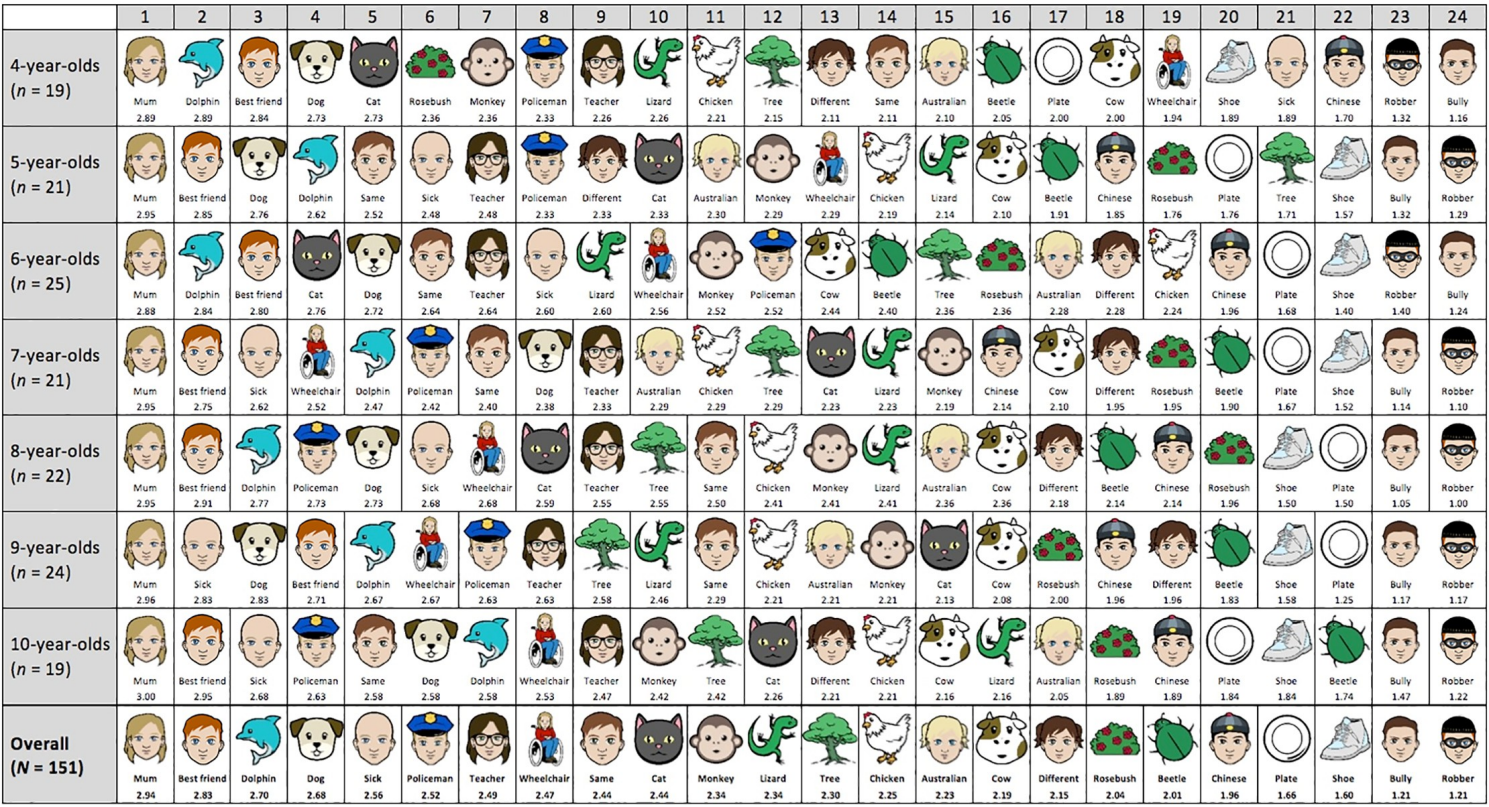
Children’s relative levels of moral concern for the 24 different entities across seven age groups (actual target stimuli depicted). The number below each target refers to a mean score, where ‘not caring at all’ responses were scored as 1, ‘caring a little’ responses were scored as 2, and ‘caring a lot’ responses were scored as 3 (range of possible means = 1–3). This is for ease of comparison only, as the ordinal logistic GLMMs treated these responses as non-linearly related categories. Note that targets with equal mean scores for a given age group are included in the same frame. Note also that the ‘best friend’ stimulus was either male or female, depending on how the child answered the question “is your best friend a boy or a girl?”.

The Age x Entity interaction (from the *a priori* cumulative logistic analysis) was followed up in a number of ways. First, we calculated simple age slopes for each of the 24 entities, in order to examine how children’s levels of moral concern towards specific entities changed linearly with age. As summarized in Fig 3, the model suggests that older children cared significantly more about people in wheelchairs (*b* = .28, SE = .09, *p* = .002, 95% CI [.10, .46]), sick children (*b* = .38, SE = .11, *p* < .001, 95% CI [.18, .59]), and trees (*b* = .31, SE = .10, *p* = .002, 95% CI [.12, .49]) than younger children. On the other hand, older children cared significantly less about cats (*b* = -.29, SE = .09, *p* = .002, 95% CI [.-.47, -.10]), and plates (*b* = -.19, SE = .09, *p* = .040, 95% CI [-.36, -.01]) than younger children. No other age slopes were significant (see S5 Table). Fig 4 displays the rankings of all 24 entities from highest to lowest level of moral concern across each of the seven age groups.

Next, we examined age-based changes in children’s *relative* levels of moral concern for different categories of entities. We conducted seven planned contrasts between pairs of categories that were expected to show differences based on the established literature. Specifically, that children would hold greater moral concern for humans vs. other forms of life, family members vs. ingroup members, ingroup vs. outgroup members, revered vs. needy individuals, high sentience vs. low sentience animals, pets vs. foot animals, and objects in comparison to villains [3, 12, 13, 14, 15]. Results are summarized in Table 1. Four of the best fitting models included a fixed effect of Category only (Ingroups > Outgroups; Objects > Villains; High sentience animals > Low sentience animals; Family > Ingroups), with no evidence that children’s relative levels of concern across these categories changed with age. The other three best fitting models included an Age x Category interaction, indicating that children’s relative levels of concern across these categories (Humans vs Other life; Pets vs Food animals; Revered vs Needy) changed with age. Comprehensive details, including the model selection procedure and the entities included in each analysis, are reproduced in the Supporting Information (S1 File).

**Table 1:** Summary of planned contrast results.

Contrast	Best Model	Category effect (overall)	Category effect (younger)	Category effect (older)
Ingroups vs. Outgroups	Category	Ingroups > Outgroups (*b* = 1.07 [.72, 1.43]; *p* < .001)	-	-
Humans (no villains) vs. Other life	Age, Category, Age x Category	-	Humans > Other (*b* = .20 [.01, .38]; *p* = .037)	Humans >> Other (*b* = .57 [.40, .75]; *p* < .001)
Villains vs. Objects	Category	Objects > Villains (*b* = 1.98 [1.53, 2.43]; *p* < .001)	-	-
High sentience animals vs. Low sentience animals	Category	High > Low (*b* = 1.20 [.84, 1.55]; *p* < .001)	-	-
Pets vs. Food animals	Age, Category, Age x Category	-	Pets >> Food (*b* = 1.75 [1.25, 2.24]; *p* < .001)	Pets > Food (*b* = 1.00 [.57, 1.43]; *p* < .001)
Family vs. Ingroups	Category	Family > Ingroups (*b* = 2.91 [2.36, 3.46]; *p* < .001)	-	-
Revered vs. Needy	Age, Category, Age x Category	-	Revered > Needy (*b* = .24 [-.19, .66]; *p* = .274)	Needy > Revered (*b* = -.40 [-.86, .06]; *p* = .085)

Note. For models including the Age x Category interaction, the main Age and Category effects were not interpreted. 95% confidence intervals are indicated in square brackets.

### Donation behavior

**Fig 5:**
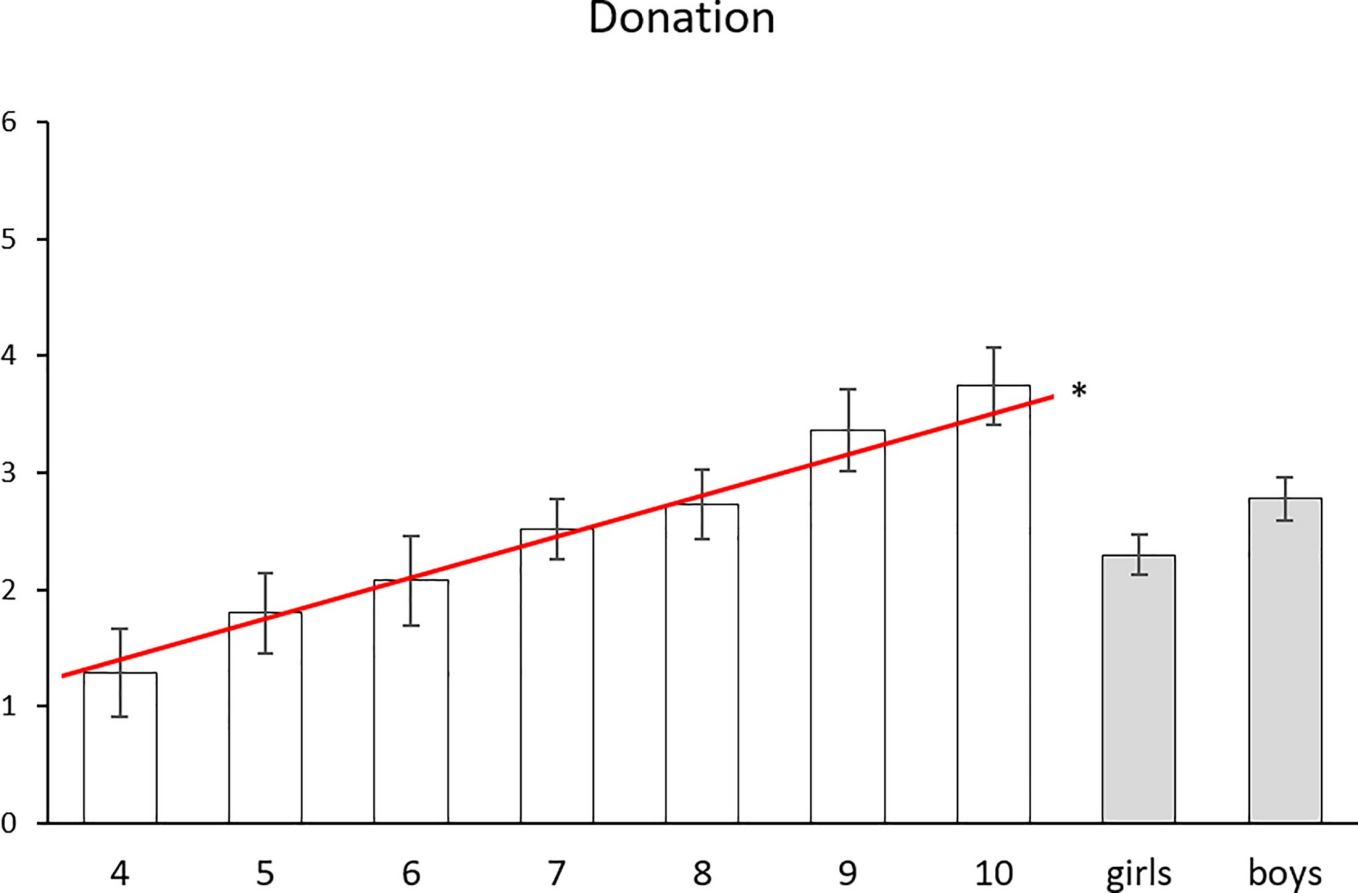
Children’s donation scores (out of 6) as a function of age and gender. The red line indicates the significant positive linear effect of age. There was no significant effect of gender.

Finally, we examined how children’s donation behavior varied as a function of the hypothesized predictors. For the purposes of these analyses, moral concern was operationalized as a continuous variable (1: did not care at all, 2: cared a little, 3: cared a lot), as this allowed us to predict donation behavior as a function of the mean moral concern score across a number of relevant entities. The number of stickers that children donated (continuous DV: 0–6) varied positively and significantly with age, *r* (144) = .450, *p* < .001, with older children donating more than younger children. A univariate ANCOVA controlling for age indicated that boys (*M* = 2.78, *SE* = .18) donated more stickers than girls (*M* = 2.30, *SE* = .17), but this effect did not reach significance, *F* (1, 142) = 3.65, *p* = .058, *η*_*p*_^*2*^ = .025. Donation behavior did not significantly vary with average level of moral concern across all entities when controlling for age, *r* (142) = .089, *p* = .287; nor did it significantly vary with average level of moral concern when this variable was binary (0: did not care at all, 1: cared at least a little), *r* (142) = .093, *p* = .267. However, donation behavior was positively and significantly predicted by the mean level of moral concern towards (non-villain) human entities, even when controlling for age, *r* (142) = .170, *p* = .042, but not the mean level of concern towards other life entities *r* (142) = .024, *p* = .774 (Fig 5). Donation behavior was not significantly predicted by the mean level of moral concern towards the needy, *r* (142) = .114, *p* = .173, or the revered, *r* (142) = .147, *p* = .079, when controlling for age.

## Discussion

Previous research with adults has shown that individual differences exist in the scope of our moral boundaries, and that such differences capture a unique element of our moral cognition [3]. The current research provides the first empirical exploration into the origins of moral expansiveness in childhood, and its development across the early human lifespan. Consistent with adult samples [3], we found no overall age-based moral expansiveness across development. However, although children are not increasing the total level of moral concern extended across all entities, with age they are more likely to perceive entities as worthy of at least basic moral consideration. Framed another way, as children age they are less willing to cast-out entities entirely from their moral circles, and make graded distinctions between levels of concern as opposed to viewing moral inclusion as a binary choice (i.e., in vs. out). This finding is in line with the original conceptualization of moral expansiveness as graded and multifaceted [3].

This pattern of results, when considered alongside evidence that moral expansiveness is driven by shifts in cultural attitudes [2], is consistent with the assertion that children, as they age, *learn* to be more morally expansive than they are otherwise predisposed to be [30]. Beyond this, though previously untested to our knowledge, it is consistent with the existence of a moral/compassion quota; that if there are limits to the scope of moral consideration we are able to extend, the structure of moral circles may fluctuate to accommodate for changes in perceptions of basic moral worth. Meaning that as we grow and acknowledge the moral standing of a greater number of entities, the amount of consideration and care we feel we can extend to individual entities may become diluted. Further, girls possess more expansive moral circles than boys, and human-related MES responses predict children’s willingness to donate rewards to an unknown peer, affirming a link between moral reasoning and prosocial behavior in development [13,18,31].

Beyond general effects of moral expansiveness, we reveal telling differences in children’s perceptions of the relative moral standing of different groups. For example, children feel greater moral concern towards members of their ingroup than their outgroup, perceive high-sentience animals (e.g., dolphins) as deserving greater moral standing than low-sentience animals (e.g., beetles), and feel villains (e.g., bullies) are less worthy of moral consideration than non-living objects (e.g., shoes). These findings reflect those established in adult samples [3], suggesting that such patterns of moral regard are stable and robust across the human lifespan. Similarly, these findings converge with established patterns of children’s moral decision making, such as the distinct moral preference for ingroup members versus outgroup members on a range of tasks [26,27], and the tendency to avoid and punish antisocial agents [32,33].

Across ages, children extend greater moral concern to humans compared to other forms of life, and to traditional pet animals versus food animals. With age, however, the distinction between the moral standing of humans versus nonhumans increases, whereas the distinction between pets and food animals decreases. Further research is required to uncover what is driving these changes, but beyond the potential impact of biological changes, such as the association between abstract thinking and reasoning and the pre-frontal cortex [34,35], one possibility is that with age children increasingly learn to regard humans as ‘special’ and nonhuman animals as a homogenous ‘other’ [36–38]. Concurrently, the acknowledgement of mental capacities in both pets and food animals [39] may begin to blur the distinction between these groups, thereby reducing the discrepancy in perceived moral worth.

Older children are also more likely to recognize the moral standing of entities in need (e.g., the sick and disabled), both in absolute terms, and in comparison to authority figures (e.g., police and teachers), than younger children. Again, further research is required, but this shift may be driven by older children embracing prescriptive norms about caring for weaker members of society, or it may emerge alongside increasing abilities to perspective-take and identify with the plight of others [20,32]. Finally, older children possess a greater sense of moral concern toward the environment (e.g., trees), and hold lesser regard for objects (e.g., plates) than younger children. This pattern may be driven by children’s increased ability to distinguish between life and non-life with age [40,41], and their general tendency to assign more moral concern to living vs. non-living entities [42]. These findings align with literature suggesting that older children increasingly display more nuanced moral reasoning skills [13–15,20,21,43–45]. Taken in sum, these findings indicate that, as children age, their structure for moral priority more closely mirrors that seen in adults [3].

In its original sense, moral expansiveness refers to the widening of moral boundaries across the course of human history [2], in a process that has accelerated in recent centuries and decades [1]. These changes to our moral circles are bound to perpetually updating cultural attitudes and practices, and thus may be said to be occurring along a cultural-level temporal axis. Here we have provided the first evidence for a second temporal axis of moral expansiveness–that involving the widening of moral boundaries across the course of the human lifespan. We note, however, that any changes along this individual-level axis will inevitably be grounded in a specific cultural framework. One would certainly not expect children growing up in medieval Europe or in a contemporary hunter-gatherer society, for example, to show the same developmental trajectories as the children in our sample [46–51]. Future studies may examine how changes along these two axes of moral expansiveness interact, and whether, for example, children from different cultural groups have varying rates of moral expansiveness with age.

Further, although the adapted moral expansiveness measure was designed to overcome methodological limitations of past research (i.e., reliance on complex language-based moral vignettes), the use of *care* as a suitable proxy of *moral* consideration might be interpreted as a limitation of the current research. Given our central aim of examining moral circle decision-making from early in development, *care* was judged to be a salient, socialized concept for young children that–in our opinion–most closely reflects concern and responsibility for the welfare of others (i.e., parents might often instruct and encourage children to ‘care’ for their pets and siblings). However, we acknowledge a level of subjectivity is likely present in participants’ interpretation of the term, and we cannot explicitly rule out that in addition to *care*, younger children may have structured entities based on familiarity and/or likeability. We do feel the emergence of the predicted relationship between our measure of moral expansiveness and prosociality, together with the fact that children reliably used the word “care” when defining each circle during our comprehension checks provide evidence in support the selection of *care* as the most suitable proxy for *moral* consideration. However, the current results should be interpreted with this potential limitation in mind.

The current research is the first to address children’s moral reasoning at an abstract level using distal, hypothetical agents as opposed to proximal characters. The results present a range of unique insights into the development of moral expansiveness and its links to prosocial behavior, demonstrating that age is but one of many contributing factors in children’s moral judgments. The simplicity and accessibility of the adapted MES makes it a novel methodological tool for examining moral concern across broad developmental trajectories. Pursuing a greater understanding of the structure and progression of boundary decision making across the human lifespan facilitates a more comprehensive understanding of the development of moral reasoning. Extending beyond theoretical contributions, furthering our knowledge of when such preferences and biases emerge is the first step in uncovering how such biases might be overcome.

## Supporting information

S1 File Supporting information. (DOCX)

S1 Table Comparison of all possible models including main effects only (cumulative logistic analysis). (DOCX)

S2 Table Comparison of previously selected model and new models including interactions (cumulative logistic analysis). (DOCX)

S3 Table Comparison of all possible models including main effects only (post hoc binomial analysis). (DOCX)

S4 Table Comparison of previously selected model and new models including interactions (post hoc binomial analysis). (DOCX)

S5 Table Age slopes for all twenty four entities. (DOCX)
