# Supplementary material for: The developmental origins of moral concern: An examination of moral boundary decision making throughout childhood
Source: PLoS One. 2018 May 29;13(5):e0197819. doi: 10.1371/journal.pone.0197819 (PMC5973598; doi:10.1371/journal.pone.0197819)
Supplement: S1 File — (DOCX) [file pone.0197819.s001.docx]

**Supporting Information**

**Target Stimuli**

Following the categories used in Crimston, Bain, Hornsey & Bastian (2016) (1), we employed two entities thought to be highly familiar to children living in the target population: Family/friends (Mum; Best friend (male or female)), In-group (Boy in Same Class; Girl from Australia), Revered (Policeman; Teacher), Stigmatized (Someone in a Wheelchair; Someone who is Very Sick), Outgroup (Girl in Different Class; Boy from China), Animals- High Sentience (Dolphin; Monkey), Animals -Low Sentience (Lizard, Beetle), Environment (Tree, Rosebush), and Villains (Robber; Bully). Instead of also including a plants category as Crimston and colleagues’ (2016) did, we provided a new category: Objects (Shoe; Plate). The inclusion of this aimed to distinguish between children’s moral consideration towards living (Environment) and non-living items (Objects).

**Cumulative Logistic GLMM: Model Selection Procedure**

Separate models containing all possible combinations of main effects (as well as a null model containing no fixed effects) were first tested against each other on the basis of AIC value (2). All models also contained a random intercept at the participant level. The best performing of these models contained significant effects of Entity and Gender, but no effect of Age (see Table S1 for summary).

All possible two-way and three-way interactions were then added to the model one at time, and these were compared to the previously selected model. Two of the new models had lower AIC values than the original model (see Table S2 for summary).

In the final step, we entered both of the model-improving interactions (Age x Entity and Age x Entity x Gender) into the same model, which gave an AIC value of 6026.27. Since this value was higher than the simpler model containing the Age x Entity interaction only (6005.47), we retained the simpler model (model 9) as the final model. As stated in the main text, this final model contained significant fixed effects of Entity, *F* (23, 3415) = 42.59, *p* < .001, Gender, *F* (1, 3415) = 6.13, *p* = .013, and Age x Entity, *F* (24, 3415) = 3.21, *p* < .001, as well as a significant random intercept, *b* = .71, SE = .11, *Z* = 6.32, *p* < .001.

**Post Hoc Binomial GLMM: Model Selection Procedure**

Separate models containing all possible combinations of main effects (as well as a null model containing no fixed effects) were again tested against each other on the basis of AIC value. Again, all models also contained a random intercept at the participant level. The best performing of these models contained significant effects of Age and Entity, as well as a non-significant Gender effect (see Table S3 for summary).

All possible two-way and three-way interactions were then added to the model one at time, and these were compared to the previously selected model. Model 9 was the only model with a lower AIC value (2580.51) than the original model (see Table S4 for summary), and we therefore retained model 9 as the final model. This final model contained significant fixed effects of Age, *F* (1, 3416) = 14.35, *p* < .001, Entity, *F* (23, 3416) = 26.28, *p* < .001, and Age x Entity, *F* (23, 3416) = 2.31, *p* < .001, a non-significant effect of Gender, *F* (1, 3416) = 3.04, *p* = .081, and a significant random intercept, *b* = 1.09, SE = .20, *Z* = 5.50, *p* < .001.

**Age Slopes for each Entity**

Table S5 displays the age slopes for all 24 entities (based on the a priori cumulative logistic analysis). Positive estimates indicate that older children cared more for an entity than younger children, whereas negative estimates indicate that older children care less for an entity than younger children. Five out of 24 total age slopes reached statistical significance.

**Comprehensive Planned Contrast Summary**

The following sections include full details of the planned contrasts following up the Age x Entity interaction in the *a priori* cumulative logistic analysis. We conducted seven contrasts between pairs of categories that were expected to show differences according to previous literature (see main text). For each contrast we tested five models against each other, each containing a unique combination of fixed effects nested within the following full factorial model: Age (continuous variable ranging from 4-10) x Category (1 vs. 2). For example, for the contrast between ingroups and outgroups, we tested the following five models: (i) Age, Category (ingroup vs. outgroup), and the Age x Category interaction (ii) Age and Category, but no interaction, (iii) Age only, (iv) Category only, and (v) a null model containing no fixed effects. Each model also contained a random intercept at the participant level, in order to account for individual differences in children’s levels of moral concern across all entities included in the comparison. Final models were again selected on the basis of AIC values. Models including the Age x Category interaction were followed up by estimating the relative levels of moral concern across both categories for a child aged at the mid-point of the younger (4-7 years) and older (7-10 years) halves of the sample (i.e., at 5.5 and 8.5 years).

**Planned contrast 1: Ingroups vs. outgroups**

This contrast tested children’s relative levels of moral concern for ingroups (Australian child and child from the same class) and outgroups (Chinese child and child from a different class). The best fitting model contained a significant effect of Category, *F* (1, 444) = 35.63, *p* < .001, suggesting that children care more about ingroups than outgroups, *b* = 1.073, *SE* = .180. The model contained no effects involving Age, suggesting that children do not vary in their relative levels of moral concern across these categories as they get older.

**Planned contrast 2: Humans (no villains) vs. other life**

This contrast tested children’s relative levels of moral concern for non-villain humans (mother, best friend, policeman, teacher, person in wheelchair, sick child, Australian child, child from the same class, Chinese child, and child from a different class) and non-human life (dog, cat, monkey, dolphin, cow, chicken, beetle, lizard, tree, rosebush). The best fitting model contained a significant Age x Category interaction, *F* (1, 2857) = 10.93, *p* = .001, suggesting that children’s relative levels of moral concern for humans and other life vary with age. To follow up this interaction, we estimated the relative levels of moral concern across these categories for a child aged at the mid-point (5.5 years) of the younger half of the sample (4-7 years), as well as for a child aged at the mid-point (8.5 years) of the older half of the sample (7-10 years). These estimates suggest that both younger and older children care more about humans than other life, but that the difference is greater for older children, *b* = .574, *SE* = .091, *t* = 6.32, *p* < .001, than for younger children, *b* = .198, *SE* = .095, *t* = 2.08, *p* = .037. **Planned contrast 3: Villains vs. objects**

This contrast tested children’s relative levels of moral concern for villains (bully and robber) and objects (shoe and plate). The best fitting model contained a significant effect of Category, *F* (1, 449) = 74.53, *p* < .001, suggesting that children care more about objects than villains, *b* = 1.982, *SE* = .230. The model contained no effects involving Age, suggesting that children do not vary in their relative levels of moral concern across these categories as they get older.

**Planned contrast 4: High vs. low sentience animals**

This contrast tested children’s relative levels of moral concern for high sentience animals (monkey and dolphin) and low sentience animals (lizard and beetle). The best fitting model contained a significant effect of Category, *F* (1, 451) = 42.90, *p* < .001, suggesting that children care more about high sentience animals than low sentience animals, *b* = 1.196, *SE* = .183. The model contained no effects involving Age, suggesting that children do not vary in their relative levels of moral concern across these categories as they get older.

**Planned contrast 5: Pets vs. food**

This contrast tested children’s relative levels of moral concern for domestic pets (dog and cat) and domestic food animals (cow and chicken). The best fitting model contained a significant Age x Category interaction, *F* (1, 450) = 7.22, *p* = .008, suggesting that children’s relative levels of moral concern for pets and food animals varies with age. Again we estimated the relative levels of moral concern across these categories for a child aged at the mid-points of the younger and older halves of the sample. These estimates suggest that both younger and older children care more about pets than food animals, but that the difference is greater for younger children, *b* = 1.749, *SE* = .252, *t* = 6.94, *p* < .001, than for older children, *b* = 1.001, *SE* = .217, *t* = 4.60, *p* < .001.

**Planned contrast 6: Family vs. ingroups**

This contrast tested children’s relative levels of moral concern for family (mother and best friend) and ingroups (Australian child and child from the same class). The best fitting model contained a significant effect of Category, *F* (1, 447) = 108.89, *p* < .001, suggesting that children care more about family than ingroups, *b* = 2.911, *SE* = .279. The model contained no effects involving Age, suggesting that children do not vary in their relative levels of moral concern across these categories as they get older.

**Planned contrast 7: Revered vs. needy**

This contrast tested children’s relative levels of moral concern for the revered (policeman and teacher) and the needy (person in wheelchair and sick child). The best fitting model contained a significant Age x Category interaction, *F* (1, 449) = 5.39, *p* = .021, suggesting that children’s relative levels of moral concern for the revered and needy varies with age. Once again we estimated the relative levels of moral concern across these categories for a child aged at the mid-points of the younger and older halves of the sample. These estimates suggest that younger children care (non-significantly) more about the revered, *b* = .240, *SE* = .219, *t* = 1.10, *p* = .274, whereas older children care (non-significantly) more about the needy, *b* = -.404, *SE* = .234, *t* = -1.73, *p* = .085.

**References**

1. Crimston D, Bain PG, Hornsey MJ, Bastian B. Moral expansiveness: Examining variability in the extension of the moral world. J Pers Soc Psychol. 2016;111(4):636–53.

2. Akaike H. A new look at the statistical model identification. Autom Control IEEE Trans. 1974;19(6):716–23.
