# Supplementary material for: The developmental origins of moral concern: An examination of moral boundary decision making throughout childhood
Source: PLoS One. 2018 May 29;13(5):e0197819. doi: 10.1371/journal.pone.0197819 (PMC5973598; doi:10.1371/journal.pone.0197819)
Supplement: S1 Table — (DOCX) [file pone.0197819.s002.docx]

Table S1. Comparison of all possible models including main effects only (cumulative logistic analysis).

| Model | Age | | Entity | | Gender | | AIC |
| --- | --- | --- | --- | --- | --- | --- | --- |
|  | *F* | *p* | *F* | *p* | *F* | *p* |  |
| 1 | .02 | .895 | 43.09 | < .001 | 6.10 | .014 | 6039.54 |
| 2 | .06 | .810 | 43.08 | < .001 | - | - | 6043.52 |
| 3 | .01 | .941 | - | - | 5.87 | .015 | 7522.38 |
| **4** | **-** | **-** | **43.09** | **< .001** | **6.16** | **.013** | **6037.55** |
| 5 | .03 | .858 | - | - | - | - | 7526.14 |
| 6 | - | - | 43.08 | < .001 | - | - | 6041.58 |
| 7 | - | - | - | - | 5.90 | .015 | 7520.38 |
| 8 | - | - | - | - | - | - | 7524.17 |

Note: best performing model is highlighted in bold.
