# Supplementary material for: The developmental origins of moral concern: An examination of moral boundary decision making throughout childhood
Source: PLoS One. 2018 May 29;13(5):e0197819. doi: 10.1371/journal.pone.0197819 (PMC5973598; doi:10.1371/journal.pone.0197819)
Supplement: S2 Table — (DOCX) [file pone.0197819.s003.docx]

Table S2. Comparison of previously selected model and new models including interactions (cumulative logistic analysis).

| Model | Additional interaction term | Interaction statistics | | AIC |
| --- | --- | --- | --- | --- |
|  |  | *F* | *p* |  |
| 4 | Original model (Entity, Gender effects only) | - | - | 6037.55 |
| **9** | **Age x Entity** | **3.21** | **< .001** | **6005.47** |
| 10 | Age x Gender | .19 | .830 | 6041.18 |
| 11 | Entity x Gender | 1.59 | .036 | 6046.52 |
| **12** | **Age x Entity x Gender** | **2.13** | **< .001** | **6026.07** |

Note: both models with lower AIC values than the original model are highlighted in bold.
