# Supplementary material for: The developmental origins of moral concern: An examination of moral boundary decision making throughout childhood
Source: PLoS One. 2018 May 29;13(5):e0197819. doi: 10.1371/journal.pone.0197819 (PMC5973598; doi:10.1371/journal.pone.0197819)
Supplement: S3 Table — (DOCX) [file pone.0197819.s004.docx]

Table S3. Comparison of all possible models including main effects only (post hoc binomial analysis).

| Model | Age | | Entity | | Gender | | AIC |
| --- | --- | --- | --- | --- | --- | --- | --- |
|  | *F* | *p* | *F* | *p* | *F* | *p* |  |
| **1** | **9.00** | **.003** | **27.61** | **< .001** | **3.17** | **.075** | **2597.58** |
| 2 | 9.44 | .002 | 27.61 | < .001 | - | - | 2598.71 |
| 3 | 9.65 | .002 | - | - | 3.29 | .070 | 3704.46 |
| 4 | - | - | 27.57 | < .001 | 3.59 | .058 | 2604.31 |
| 5 | 10.09 | .002 | - | - | - | - | 3705.71 |
| 6 | - | - | 27.57 | < .001 | - | - | 2605.87 |
| 7 | - | - | - | - | 3.72 | .054 | 3711.79 |
| 8 | - | - | - | - | - | - | 3713.47 |

Note: best performing model is highlighted in bold.
