# Supplementary material for: The developmental origins of moral concern: An examination of moral boundary decision making throughout childhood
Source: PLoS One. 2018 May 29;13(5):e0197819. doi: 10.1371/journal.pone.0197819 (PMC5973598; doi:10.1371/journal.pone.0197819)
Supplement: S4 Table — (DOCX) [file pone.0197819.s005.docx]

Table S4. Comparison of previously selected model and new models including interactions (post hoc binomial analysis).

| Model | Additional interaction term | Interaction statistics | | AIC |
| --- | --- | --- | --- | --- |
|  |  | *F* | *p* |  |
| 1 | Original model (Age, Entity, Gender effects) | - | - | 2597.58 |
| **9** | **Age x Entity** | **2.31** | **< .001** | **2580.51** |
| 10 | Age x Gender | .88 | .348 | 2598.70 |
| 11 | Entity x Gender | 1.25 | .188 | 2610.95 |
| 12 | Age x Entity x Gender | 1.50 | .015 | 2609.23 |

Note: both models with lower AIC values than the original model are highlighted in bold.
