# Supplementary material for: The developmental origins of moral concern: An examination of moral boundary decision making throughout childhood
Source: PLoS One. 2018 May 29;13(5):e0197819. doi: 10.1371/journal.pone.0197819 (PMC5973598; doi:10.1371/journal.pone.0197819)
Supplement: S5 Table — (DOCX) [file pone.0197819.s006.docx]

Table S5. Age slopes for all twenty four entities.

| Entity | *b* | *SE* | *t* | *p* |
| --- | --- | --- | --- | --- |
| Mum | .253 | .20 | 1.24 | .217 |
| Best friend | -.038 | .13 | -.30 | .764 |
| Policeman | .188 | .10 | 1.94 | .053 |
| Teacher | .074 | .09 | .77 | .439 |
| Person in wheelchair | .284 | .09 | 3.08 | .002* |
| Sick child | .383 | .11 | 3.64 | < .001* |
| Ingroup child^1^ | -.050 | .09 | -.57 | .571 |
| Outgroup child^2^ | .089 | .09 | 1.01 | .311 |
| Same class child | .020 | .09 | .22 | .829 |
| Different class child | -.081 | .09 | -.92 | .360 |
| Dog | -.087 | .10 | -.84 | .291 |
| Cat | -.289 | .09 | -3.07 | .002* |
| Monkey | -.031 | .09 | -.34 | .734 |
| Dolphin | -.195 | .11 | -1.84 | .066 |
| Cow | .010 | .09 | .12 | .908 |
| Chicken | .008 | .09 | .09 | .926 |
| Beetle | -.149 | .09 | -1.67 | .095 |
| Lizard | -.010 | .09 | -.11 | .910 |
| Tree | .305 | .10 | 3.18 | .002* |
| Rosebush | -.142 | .09 | -1.60 | .109 |
| Bully | .118 | .13 | .89 | .372 |
| Robber | -.245 | .15 | -1.62 | .108 |
| Shoe | .055 | .09 | .58 | .562 |
| Plate | -.185 | .09 | -2.05 | .041* |

* *p* < .05

^1^Described as an Australian child

^2^Described as a Chinese child
